# ESR Essentials: how to get to valuable radiology AI: the role of early health technology assessment—practice recommendations by the European Society of Medical Imaging Informatics

**DOI:** 10.1007/s00330-024-11188-3

**Published:** 2024-12-05

**Authors:** Erik H. M. Kemper, Hendrik Erenstein, Bart-Jan Boverhof, Ken Redekop, Anna E. Andreychenko, Matthias Dietzel, Kevin B. W. Groot Lipman, Merel Huisman, Michail E. Klontzas, Frans Vos, Maarten IJzerman, Martijn P. A. Starmans, Jacob J. Visser

**Affiliations:** 1 Department of Radiology and Nuclear Medicine, Erasmus University Medical Center Rotterdam, Rotterdam, The Netherlands; 2 Department of Medical Imaging and Radiation Therapy, The Hanze University of Applied Sciences, Groningen, The Netherlands; 3 Department of Radiotherapy, University of Groningen, University Medical Centre Groningen, Groningen, The Netherlands; 4 Research Group Healthy Ageing, Allied Health Care and Nursing, The Hanze University of Applied Sciences, Groningen, The Netherlands; 5 Erasmus School of Health Policy & Management, Erasmus University Rotterdam, Rotterdam, The Netherlands; 6 K-SkAI LLC, Petrozavodsk, Russia; 7 ITMO University, St. Petersburg, Russia; 8 Department of Radiology, University Hospital Erlangen, Erlangen, Germany; 9 Department of Radiology, The Netherlands Cancer Institute, Amsterdam, The Netherlands; 10 Department of Thoracic Oncology, Netherlands Cancer Institute, Amsterdam, The Netherlands; 11 Radboud University Medical Center, Department of Radiology and Nuclear Medicine, Nijmegen, The Netherlands; 12 Department of Radiology, School of Medicine, University of Crete, Heraklion, Greece; 13 Division of Radiology, Department of Clinical Science, Intervention and Technology (CLINTEC), Karolinska Institutet, Stockholm, Sweden; 14 Computational Biomedicine Laboratory, Institute of Computer Science, Foundation for Research and Technology (FORTH), Heraklion, Crete, Greece; 15 Department of Imaging Physics, Delft University of Technology, Delft, The Netherlands; 16 Department of Pathology, Erasmus University Medical Center Rotterdam, Rotterdam, The Netherlands

**Keywords:** Artificial intelligence, Technology assessment (Biomedical), Radiology, Value-based healthcare, Stakeholder participation

## Abstract

**Abstract:**

AI tools in radiology are revolutionising the diagnosis, evaluation, and management of patients. However, there is a major gap between the large number of developed AI tools and those translated into daily clinical practice, which can be primarily attributed to limited usefulness and trust in current AI tools. Instead of technically driven development, little effort has been put into value-based development to ensure AI tools will have a clinically relevant impact on patient care.

An iterative comprehensive value evaluation process covering the complete AI tool lifecycle should be part of radiology AI development. For value assessment of health technologies, health technology assessment (HTA) is an extensively used and comprehensive method. While most aspects of value covered by HTA apply to radiology AI, additional aspects, including transparency, explainability, and robustness, are unique to radiology AI and crucial in its value assessment. Additionally, value assessment should already be included early in the design stage to determine the potential impact and subsequent requirements of the AI tool. Such early assessment should be systematic, transparent, and practical to ensure all stakeholders and value aspects are considered. Hence, early value-based development by incorporating early HTA will lead to more valuable AI tools and thus facilitate translation to clinical practice.

**Clinical relevance statement:**

This paper advocates for the use of early value-based assessments. These assessments promote a comprehensive evaluation on how an AI tool in development can provide value in clinical practice and thus help improve the quality of these tools and the clinical process they support.

**Key Points:**

- *Value in radiology AI should be perceived as a comprehensive term including health technology assessment domains and AI-specific domains.*
- *Incorporation of an early health technology assessment for radiology AI during development will lead to more valuable radiology AI tools.*
- *Comprehensive and transparent value assessment of radiology AI tools is essential for their widespread adoption.*

## Key recommendations

- Assessment of value for radiology AI should consider all potential aspects of value. These are described by health technology assessment domains and radiology AI-specific domains such as Clinical Effectiveness, Cost-Effectiveness, Patient and Societal Impact, Explainability, and Generalisability. (Level of evidence: Moderate)
- Early health technology assessment for radiology AI should be an integral part of the development of AI tools. The method used should be systematic, transparent, and practical with consultation of stakeholders and end-users to ensure tools fit the high standards of clinical practice and address relevant needs in healthcare. (Level of evidence: Low)
- Only radiology AI tools with transparent value assessments of their prospective impact on a specifically intended workflow should be considered for widespread clinical integration. This includes the effects of the local environment (e.g. patient population, scanning protocols, patient management) on the performance of the AI tool. (Level of evidence: Moderate)

## Introduction

In recent years, an increasing number of artificial intelligence (AI) tools for radiology have been developed, showing promising performance in research settings, sometimes similar to or exceeding that of radiologists [1]. However, only a limited number of these tools are actually used in daily clinical practice, which is often due to a mismatch between the functionality of the developed AI tool and the needs of daily clinical practice. Many such tools are only evaluated for their technical performance and diagnostic accuracy, often in controlled conditions (e.g. strict inclusion/exclusion criteria), deviating from real-world practice [2–5]. Impact of the tool on diagnostic thinking, treatment decisions, clinical workload, patient outcomes, including cost-effectiveness analysis, is often not quantified or not even considered during development [2, 5–8]. A 2022 European Society of Radiology survey illustrates the mismatch between developers and end-users, which showed that around 40% of radiologists had practical clinical experience with AI-based tools, while only a little over 10% had an interest in acquiring AI for their practice [9]. Hence, there is a need for early assessment of the potential value of proposed radiology AI tools in order to enhance their value in clinical practice [10–12].

**Fig. 1:**
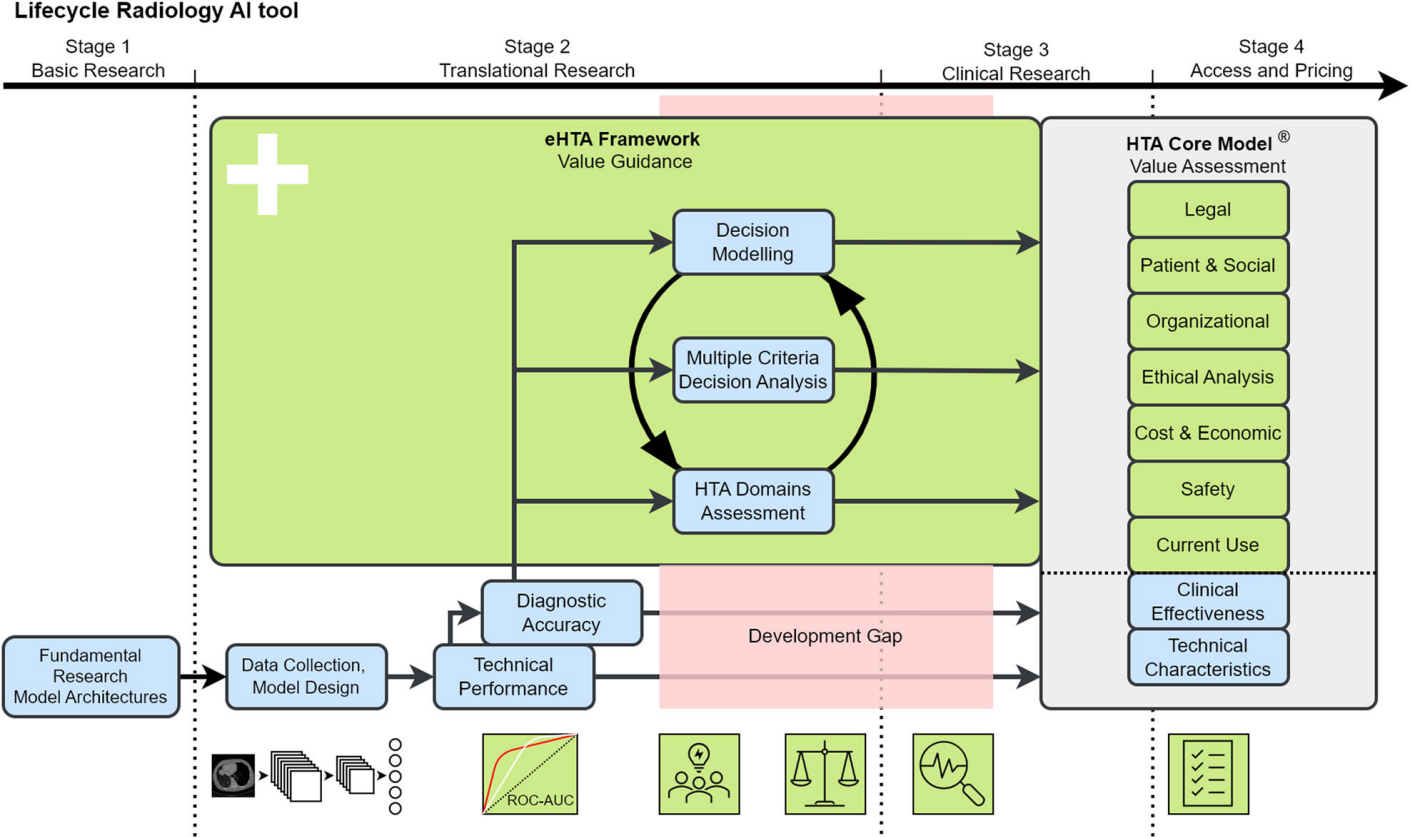
Simplified overview of the radiology AI development. Four stages as distinguished by IJzerman et al [40]: Basic Research, Translational Research, Clinical Research, and Access and Pricing. Valuable AI in radiology can be determined by comprehensive value assessment such as the Health Technology Assessment (HTA) Core Model®. Guidance during the development process to achieve value and cover the development gap can be performed by an early Health Technology Assessment (eHTA) framework visualised here as an iterative process of HTA domain assessment and two eHTA methods (e.g. decision modelling and multiple criteria decision analysis). AI, artificial intelligence; eHTA, early Health Technology Assessment; HTA, Health Technology Assessment

Early health technology assessment (eHTA) in various forms has been used in healthcare as an essential element for valuable tool development [13]. eHTA is based on the comprehensive evaluation of the health technology assessment (HTA) but shifts the moment of evaluation. Instead of evaluation based on in-clinic testing such as RCT studies, eHTA is initiated at the start of the translational research in the design phase of the tool. eHTA is based on predictions and expectations, which are updated with the latest information until a pilot or research trial has been started (see Fig. 1). This provides the opportunity to identify valuable objectives for technology early on. The eHTA by de Windt TS et al [14] for cartilage repair technologies is one such example. However, in this example the evaluation is limited to only an economic prediction model. This limited scope, therefore, misses other value aspects that are part of the eHTA. Furthermore, the unique properties of radiology AI will require additional value aspects beyond the existing eHTA methods. Currently, a comprehensive method for the eHTA of AI in radiology is missing.

The aim of this paper is, therefore, to identify how eHTA could be used in the radiology AI development to facilitate value-based AI and thereby bridge the gap between research and clinical practice (see Fig. 1). First, HTA is introduced to explain a general methodology for value assessment in healthcare, focussing on the HTA Core Model^®^. Second, we will identify the differences between value assessment for AI versus non-AI tools and the unique properties of radiology AI. The FUTURE-AI guideline and RADAR framework will be discussed as examples in adopting the HTA Core Model^®^ for Radiology AI. Third, we will describe the benefits of eHTA in the development of health technologies and how eHTA can be performed for radiology AI tools. Finally, we will present our vision for the adoption of eHTA as common practice in the development of radiology AI tools.

## Health technology assessment

### Health technology assessment in healthcare

For a general assessment of AI tools, it is of utmost importance to consider their potential value and clinical impact. Generally, “value” in healthcare is described as the measured improvement in a patient’s health outcome for the cost of achieving that improvement [15]. However, various interpretations of what measures are considered a health outcome and which costs are linked to those outcomes, have resulted in the development of multiple value assessment methods [16–18]. Such assessments are part of the HTA research field. HTA is a multidisciplinary process that summarises information about the medical, social, economic, and ethical issues related to the use of health technology in a complete, systematic, transparent, unbiased, and robust manner. The purpose of this is to provide a complete overview of the value of patient health technology.

The HTA Core Model^®^ is one of the most extensively researched methods [19]. It has become the go-to model of (European) HTA agencies for performing and reporting their recommendations for reimbursement of newly developed drugs and other health technologies and is, therefore, our main focus.

### HTA Core Model^®^

The HTA Core Model^®^ has been developed as a multidisciplinary, comprehensive value assessment framework that is relevant and applicable across a variety of projects and organisations. The HTA Core Model^®^ considers nine domains of value assessment: Current Use, Technical, Safety, Clinical Effectiveness, Cost & Economic, Ethical Analysis, Organisational, Patient & Social, and Legal. Each domain describes a different aspect of the health technology with the goal of providing a comprehensive checklist for value evaluation. The HTA Core Model^®^ does not directly provide a recommendation or value for a health technology, instead it is a systematic comprehensive approach to ensure all potential aspects of value are considered, summarised, and reported as shown by the examples of Mäkelä et al [20] and Galekop et al [21]. Not all aspects of the HTA Core Model^®^ might be relevant for a specific health technology, but the model minimises the chance that a negative or positive impact of the health technology is overlooked.

The HTA Core Model^®^ could serve as a solid basis for HTA in radiology AI, as most of its value aspects apply to the field of radiology AI. However, value assessment of AI tools also comprises several unique aspects that are not found in non-AI health technologies, which we discuss in the next section.

## Value-based AI in radiology

### Current value assessments for radiology AI tools

Value of AI tools is often mainly assessed by technical performance and diagnostic accuracy rather than on a patient-, healthcare- or societal level [16]. Conventionally, the technical performance of the model is evaluated through the stability and interoperability of the AI’s pipeline and processing speed. In the last few years, multiple reporting guidelines have been established to broaden the assessment scope and help describe the development process for radiology AI tools (e.g. Decide-AI, CLAIM, CLEAR) [22–28]. These guidelines enhance the transparency in the method of AI development and serve as a checklist for developers to ensure certain tasks have been performed [23]. However, since these are reporting guidelines, they lack a systematic and practical approach useful for AI model development.

### AI versus non-AI

There are key differences between AI and other non-AI health technology tools (see Table 1). Instead of performing pre-determined operations, as is typically implemented in non-AI methodology, AI tools automatically learn patterns from example data. As such, AI can combine data from various sources and reveal complex relations to support and renew our insight into diagnostics, prognostics, and therapy choices. Frequently, however, the highly complicated data processing underlying AI tools comes with a lack of transparency into their exact working mechanisms [29–31]. These AI-specific issues mean that the general HTA Core Model^®^ falls short in the value assessment of radiology AI tools [32, 33].

**Table 1:** Health technology assessment relevant differences between AI and non-AI health technology tool characteristics

Characteristic	Non-AI	AI
Technical design of the model	Human defined logic	Self-learned logic
Knowledge distillation from data	Pre-defined interactions	New complex interactions
Explainability of the model	Relatively transparent	More black-box like

### FUTURE-AI guidelines for trustworthy and deployable AI

An excellent source for potentially extending the HTA Core Model^®^ is the Delphi-consensus-based FUTURE-AI framework for trustworthy and deployable AI [34]. The framework has a comprehensive value scope, comparable to HTA, but the value concepts are specified for AI in radiology. FUTURE-AI provides 28 guiding principles based on six guiding key-concepts of trustworthy and deployable AI in healthcare: Fairness, Universality, Traceability, Usability, Robustness, and Explainability. Since a wide variety of definitions exist for these concepts, we highlight some supplementation examples of FUTURE-AI concepts for different HTA domains.

The main purpose of the HTA domain “Current Use” is to provide a description of the target conditions and current management of those conditions with a medical focus (e.g. epidemiology, overdiagnosis). FUTURE-AI’s first guiding principle for Universality is to define intended use and user requirements. The guiding principle adds emphasis on the variations between healthcare institutions that can impact the generalisability of an AI tool (e.g. target population, medical equipment, or IT infrastructure).

The ethical analysis described by the HTA Core Model^®^ considers prevalent social and moral norms and values relevant to the technology in question. The domain covers nineteen issues, which will identify most of the AI-related ethical issues, such as potential input biases. FUTURE-AI considers the tendency of AI tools to identify unethical correlations, even though the input data seemed balanced. This Fairness aspect, therefore, adds monitoring methods to detect and mitigate biases arising from the AI tool.

Value-for-money judgments, as covered by the “Cost & Economic” domain of HTA, summarise the evidence from the “Safety” and “Effectiveness” domains into one transparent, structured cost-benefit analysis. Summarising all the costs and benefits of a radiology AI tool can be challenging as the influence of the tool can be complex. The stakeholder impact assessment, part of the FUTURE-AI Usability, promotes end-user interaction and helps identify AI-specific impacts for each stakeholder, resulting in a more accurate cost-benefit analysis.

The HTA Core Model^®^ falls short in the value assessment of radiology AI tools. As such, the key-concepts as described by FUTURE-AI can be used to supplement the HTA domains, adding them to the Core Model checklist to ensure all potential aspects of value are considered at least once. An example of a value evaluation methodology roughly incorporating the value domains of both the HTA Core Model^®^ and the FUTURE-AI guideline is the hierarchically structured RADAR framework [35].

### RADAR framework for value assessment of radiology AI

**Fig. 2:**
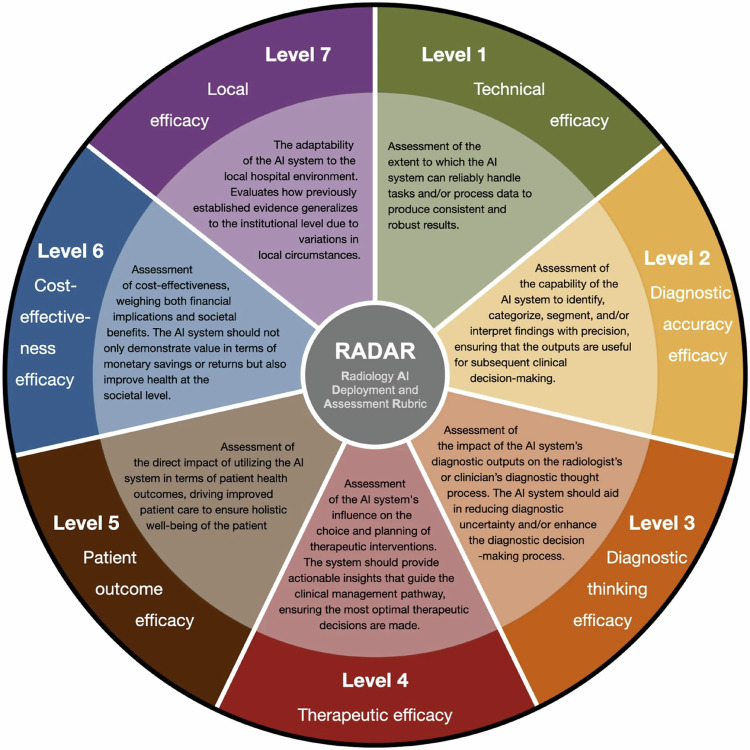
Overview of the Radiology AI Deployment and Assessment Rubric (RADAR) framework, which could form a basis for early health technology assessment in radiology AI. The outer circle depicts the RADAR efficacy level, and the inner circle provides its description. AI, artificial intelligence. Reproduced from [35] Boverhof BJ et al (2024). Licensed under CC BY 4.0 https://creativecommons.org/licenses/by/4.0/

The RADAR framework incorporates the lifecycle of a diagnostic imaging tool and is organised in seven hierarchical levels similar to those of the Fryback model [36] and according to increasing degrees of evidence (see Fig. 2). The hierarchical levels of the RADAR framework reflect a broad scope of assessment as also described by HTA and FUTURE-AI, while simultaneously indicating the logical path for evidence growth of the value of a technology. For instance, if an AI tool is not reliable (level 1) or not accurate in its predictions (level 2), then there is little use in calculating the cost-effectiveness of such an AI tool (level 6).

Another important difference between the RADAR framework and HTA Core Model^®^, is their intended user. HTA is mainly developed to inform policymakers. Therefore, it is more explicit in summarising all aspects of value as available at one timepoint. The RADAR framework is more focused on the developer and end-user, e.g. the radiologist, providing a blueprint on how to organise the available studies on the effectiveness of the tool and what types of studies still need to be performed to complete a value assessment. Eventually, this hierarchical structure might, however, result in a less comprehensive evaluation of value compared to the HTA Core Model^®^. RADAR is a recently developed framework: studies conceptualised based on RADAR have not yet been performed.

For both the HTA Core Model^®^ and RADAR framework, evidence of the value of a tool is collected over time by different studies. As described by the RADAR levels, one starts with technical performance, eventually proceeding towards the higher valued levels of evidence. Incidentally, a major issue or obstacle identified at a higher level can make the designed AI tool ineffective or impractical. Therefore, it can be useful to shortly consider each level at least once at the beginning of the development of a new tool by performing a light scan of potential issues, which is the premise of eHTA.

## Early health technology assessment

### The function of eHTA

Technical performance and diagnostic accuracy measures (e.g. sensitivity and specificity) are not only used in evaluation after the development of AI tools but also during the development and training of the AI tools. Generally, optimisation of a model on only these diagnostic measures has been deemed sufficient to result in a valuable tool [2, 37]. However, it has become apparent that these measurements provide an inadequate reflection of the value of the tool [4]. For example, while an AI tool might have the same diagnostic accuracy as standard care and would therefore appear to offer no added value, it could be optimised to reduce radiologist assessment time or improve healthcare efficiency [38]. Therefore, it is important to consider, early on, how the overall value of an AI tool will later be determined by purchasers and procurers.

If we know beforehand how the value of an AI tool will be assessed later, e.g. by HTA, we might use this insight to assess the potential value of the tool during development. This concept of an “early value assessment” has been acknowledged as a useful method for timely scrutinising and updating the objectives and properties of the technology to explore its potential value [39]. Developers may conduct such an assessment at any time during development, from the initial design concept until first evaluations in the clinical practice [40, 41]. While an early value assessment such as eHTA is useful for developers in updating the technology’s objectives and design, its output can support decisions by other stakeholders regarding resource allocation, clinical trial design, and pricing strategies [13, 42]. Furthermore, results from eHTA can be used as input for HTA after clinical deployment (see Fig. 1).

### eHTA for radiology AI

An eHTA for radiology AI should be systematic, transparent, and practical. Systematic analysis provides a way to perform quality control [43], facilitating comprehensive evaluation of all valuable domains for radiology AI. A systematic approach also ensures reproducibility, which promotes the trustworthiness of the outcome [44]. For the developer, transparency in the eHTA method can facilitate and accelerate decision-making during tool development [45]. Furthermore, if the eHTA is transparent, relevant stakeholders (e.g. investors, policymakers, radiologists, other physicians, ethicists, legal experts, health insurers, and patients) can more easily understand why and how certain decisions were made during development, which enhances trust in the radiology AI tool [23]. Lastly, practical integration of the eHTA into the development process is essential [44]. Seamless integration into the development process and active participation of end-users (e.g. clinicians, financial suppliers, and patients) is crucial for performing a meaningful assessment. As AI development is often a fast-paced agile process, the eHTA should facilitate this with rapid, repeated evaluations, likely making it an iterative process [46].

### Methods for eHTA

There is no single standard method to perform an eHTA [47, 48]. Various methods have been used for eHTA in other medical fields [49–51]. Ultimately, the most appropriate eHTA method depends on the objectives of the assessment and characteristics of the technology (e.g. intended use, required hardware) [48]. In addition, a mix of different approaches may be used to assess various aspects of value for the technology in development [40]. An eHTA for radiology AI aims to support defining valuable objectives and designs for the AI tool in development. Decision-making methods such as multiple criteria decision analysis (MCDA) are particularly suitable for this objective [40, 52]. These decision-making methods involve a comparison of the value of potential tool alternatives, which can be used to define objectives and tool designs that incorporate those AI tool aspects deemed more valuable.

MCDA is a versatile eHTA method [53] for systematic [54] and transparent [55] decision-making. The method first focuses on assembling criteria influencing the value of a radiology AI tool. This is done by identifying differences between potential alternate designs for the reviewed AI tool. Stakeholder elicitation is used to assign potential performances of the alternate designs and weights of importance for each criterion [56]. The result is a comprehensive overview of value-influencing factors that can be used to support design choices. Guidelines are available that describe how to assemble criteria, select an analysis method for weight assessment performances approximating, and aggregate the value scores [54]. The reliability of the MCDA results is highly dependent on the selected analysis methods [53]. Furthermore, interaction with stakeholders is crucial to define meaningful criteria, weights, and performances. It can be difficult to perform a good MCDA since it requires a representative set of stakeholders and could be both costly and time-consuming. However, a correctly performed MCDA is a powerful tool to achieve a transparent value overview supporting the design choices of AI tools or medical technologies, as shown by Hilgerink et al [57].

In addition to MCDA, decision modelling can be used to evaluate the potential value (cost-effectiveness in this context) of new technologies by simulating various scenarios, such as the accuracy of the AI algorithm, to predict impacts on health outcomes and costs. Examples of decision models for cost-effectiveness are provided by Marka et al [58] and Buisman et al [41]. These scenario analyses can ensure seamless and cost-effective integration of innovation into existing healthcare systems by providing valuable insights for possible clinical pathway integrations, feasible pricing, and required performance levels [59]. However, currently, eHTA methods such as MCDA and decision modelling are rarely performed, emphasising a gap in the development cycle, which may be the basis of why the practical application of AI tools remains limited.

### Future vision for eHTA in radiology

AI in radiology is not exceptional; a comprehensive value assessment should be performed as is common with other health technologies. Furthermore, post-development evaluation will not guarantee the development of valuable AI tools. Therefore, performing HTA and eHTA methodologies should become common practice for introduction of any radiology AI tool.

Still, adopting an eHTA standard is challenging. First, performing an eHTA needs to be feasible for developers. This requires an eHTA process to be flexible and iterative to adapt to design updates and fit in the development process. Furthermore, the selected eHTA methods should be generalisable to remain useful for new developments in medical technology, especially for the fast-evolving field of AI. Third, the cooperation of a broad set of stakeholders is required for a successful eHTA process.

The first two challenges can be addressed with the development of a standardised framework for radiology AI-specific eHTA. Significant effort has been placed in the development of comprehensive HTA methodologies for healthcare such as the HTA Core Model^®^, but not specifically for radiology AI [19]. The unique properties of AI and radiology require adoptions of the general HTA strategies, which may be based on existing guidelines and frameworks for deployable radiology AI (i.e. FUTURE-AI and RADAR). Alternatively, inspiration can be gained from other research fields focused on optimising the technology development process, such as model-based systems engineering [60]. A standardised framework can streamline the eHTA process. Still, time and resources might not accommodate performing a full eHTA, for which a set of minimal requirements can serve as an alternative.

The eHTA framework introduces an additional issue since the lack of validation studies requires the process to be functional with predictions. The proposed HTA framework for radiology AI and existing eHTA methodologies [54] can be combined into a standardised, detailed, and feasible eHTA process addressing radiology AI specifics and use methods designed for predictive modelling. Standardisation of the eHTA process will, besides improving the adaption of eHTA, also improve the possibility to share and compare eHTA reports between institutions as is done for the HTA Core Model^®^.

For the third challenge, end-users (e.g. radiologist, patients) play a vital role in the development and use of these radiology AI-specific HTA and eHTA frameworks. Only when end-users expect and request transparent and comprehensive evaluations will they become common practice. This can be accelerated by the active participation of end-users in establishing the requirements since this development process should be collaborative and multidisciplinary.

The major gap between the large number of developed AI tools and those translated into daily clinical practice has to be addressed by the systematic, transparent, unbiased, and robust manner an eHTA for radiology AI provides. This will require community effort to define standardised eHTA methods how comprehensiveness, transparency and bias can be measured. However, it starts with perceiving value in radiology AI as a comprehensive term and incorporating the eHTA for radiology AI in the development process of new radiology AI tools.

## Summary statement

Value assessment is not something new. Comprehensive frameworks like the health technology assessment (HTA) Core Model^®^ are standard practice for most healthcare technologies. Radiology AI is not exempted from value assessment and thus should be evaluated like other health technologies. To address the unique value characteristics of AI in radiology, the HTA value domains can be supplemented with radiology AI-specific domains such as those identified by FUTURE-AI.

For effective development of valuable AI in radiology, evaluation of value should be an integral part of the development process rather than an evaluation after development. With an early HTA (eHTA) the value aspects of a HTA are evaluated with methods capable of handling the limited and often predictive data, such as MCDA and decision modelling.

To promote the adoption of eHTA during the development of radiology AI tools, a standardised and practical eHTA framework still needs to be integrated into the development process. Furthermore, collaboration with the clinic in the development of new radiology AI tools is required. Active participation of a broad group of stakeholders should be included during the development and transparency in this process should be requested by all end-users of new AI tools. The eHTA value-based assessment is recommended as a method to minimise the gap between a developed AI tool and its effective in-clinic use. Although a full eHTA might not be feasible for every newly developed AI tool, increased transparency in the development process before entry into the market will result in more valuable AI tools.

## Patient summary

Artificial intelligence (AI) tools for radiology are expected to contribute to the revolution of healthcare. However, radiologists who used AI tools have not yet been convinced of their health improving capabilities. One of the main issues is the limited consideration of the actual value of a tool during development. Early evaluation of the value of a tool should be part of every radiology AI tool in development and should consider all persons who might be impacted by it. Only with such a strategy will new AI tools really enhance radiology.

## Abbreviations

eHTA: Early health technology assessment
HTA: Health technology assessment
MCDA: Multiple criteria decision analysis
